# Changes in Intestinal Microbial Community of the Black Tiger Shrimp *Penaeus monodon* in Response to Triclocarban Exposure

**DOI:** 10.3390/biology14111542

**Published:** 2025-11-03

**Authors:** Yafei Duan, Yuxiu Nan, Jianhua Huang, Zhe Zhang, Yanming Sui, Xueming Dan

**Affiliations:** 1 State Key Laboratory of Mariculture Biobreeding and Sustainable Goods, Key Laboratory of South China Sea Fishery Resources Exploitation & Utilization, Ministry of Agriculture and Rural Affairs, South China Sea Fisheries Research Institute, Chinese Academy of Fishery Sciences, Guangzhou 510300, China; 2 Shenzhen Base of South China Sea Fisheries Research Institute, Chinese Academy of Fishery Sciences, Shenzhen 518121, China; 3 College of Marine and Biological Engineering, Yancheng Institute of Technology, Yancheng 224051, China; 4 Lianyungang Xuanda Biotechnology Co., Ltd., Lianyungang 222100, China

**Keywords:** shrimp, triclocarban, hazardous substances, intestinal microbiota

## Abstract

Triclocarban (TCC), a synthetic antimicrobial compound prevalent in personal care products, has emerged as a typical contaminant in aquatic ecosystems. Intestinal microbiota maintains the host’s health homeostasis by regulating nutrient metabolism and immunity and is regarded as a sensitive biomarker for the risk assessment of pollutants. Currently, there is still a lack of toxicity assessment of TCC on the intestinal microbiota homeostasis of shrimp. Therefore, this study employed 16S rDNA sequencing to explore intestinal microbiota perturbations in *Penaeus monodon* following subchronic exposure (14 days) to graded TCC concentrations (1 and 10 μg/L). The results showed that TCC exposure altered intestinal microbiota diversity, marked by increases in the ACE, Chao1, and Shannon indices and a decrease in the Simpson index; however, none of these changes reached statistical significance (*p* > 0.05). Furthermore, the community composition was also altered, characterized by a significant increase in Bacteroidetes and a significant decrease in Tenericutes (*p* < 0.05), alongside non-significant increases in Proteobacteria and decreases in Firmicutes (*p* > 0.05). The abundances of some putative beneficial bacterial genera (*Alloprevotella*, *Bacteroidales S24-7 group_norank*, *Cetobacterium*, *Enterococcus* and *Lactobacillus*) and harmful bacteria (*Photobacterium* and *Aeromonas*) were decreased (*p* > 0.05); the abundance of *Vibrio* was decreased in the T1 group but increased in the T10 group (*p* > 0.05). Additionally, the predicted functions of the intestinal microbiota, such as glycan biosynthesis and degradation, steroid and isoflavone biosynthesis, and nucleotide metabolism, were enhanced. These results indicated that TCC exposure had a negative effect on the homeostasis of the intestinal microbiota of *P. monodon*.

## Simple Summary

Triclocarban (TCC), an antimicrobial agent previously widely used in personal care and health products, poses potential environmental contamination risks. The intestinal microbiota is closely associated with host health and remains sensitive to pollutant exposure. This study investigated the toxic effects of TCC on the intestinal microbiota of an edible black tiger shrimp, *Penaeus monodon*, by exposing them to 1 and 10 μg/L TCC for 14 days. The results indicated that TCC exposure increased intestinal microbiota diversity, though the change was not statistically significant. In terms of community composition, TCC led to a significant increase in Bacteroidetes and a significant decrease in Tenericutes. The abundances of both potentially beneficial bacteria and opportunistic pathogens showed decreasing trends, although these changes were not statistically significant. Functional prediction analysis revealed significant enhancements in metabolic pathways related to the biosynthesis and degradation of glycans, steroids, isoflavones, and other substances. These alterations demonstrate that TCC exposure disrupts the homeostatic balance of the intestinal microbiota in *P. monodon*, suggesting that this pollutant may pose a potential threat to aquatic organism health through its impact on the intestinal microbiota.

## 1. Introduction

Triclocarban (TCC) is a broad-spectrum antimicrobial agent widely used in personal care products, medical devices, and textiles [1]. Due to its high stability and persistence, TCC has become a typical trace organic pollutant in global aquatic environments [2]. In recent years, environmental monitoring data from various regions worldwide have detected TCC residues in rivers, lakes, coastal waters, and effluents from wastewater treatment plants [3]. For example, TCC concentrations have been detected in the Maryland river (6.75 μg/L) [4], in the Indian river (1.12 μg/L) [5], and in the Shijing river (338 ng/L), and part of the Pearl River system in South China [6]. Previous studies have confirmed that TCC can disrupt the endocrine system, inhibit antioxidant capacity, induce apoptosis, and cause significant impairments to reproductive development and immune function in aquatic species. For instance, TCC at 0.1 and 6.3 µg/L can disrupt embryonic development in the silver catfish (*Rhamdia quelen*) [7]. Exposure to 560 ng/L TCC can affect the body length of juvenile fathead minnow (*Pimephales promelas*) [8]. A concentration gradient of TCC (1, 10, 100 μg/L) has been found to interfere with lipid metabolism in the black-spotted frogs (*Pelophylax nigromaculatus*) [9]. Moreover, TCC at 1 and 10 μg/L impacts the gill histology, induces stress responses, and disrupts physiological homeostasis in the black tiger shrimp (*Penaeus monodon*) [10].

The intestinal microbiota is often regarded as a critical “micro-organ” in animals, playing a central role in regulating the nutrient absorption, energy metabolism, immune defense, and detoxification of the host [11,12]. When exposed to pollutants, the intestinal microbiota will respond rapidly, and its community changes are frequently used as sensitive biomarkers for assessing the toxicity of contaminants [13]. Furthermore, a disrupted intestinal microbiota can adversely affect their host health, increasing susceptibility to diseases [14,15]. For example, in mice, TCC exposure can induce intestinal toxicity by disrupting the microbial community and triggering AhR-mediated inflammation [16]. In zebrafish (*Danio rerio*), TCC exposure can reduce intestinal microbial diversity and alter community composition, directly leading to impaired intestinal health [17]; this microbiota dysbiosis can further impair the gut–brain-gonad axis, and ultimately impair the zebrafish’s growth and development [18]. Notably, the biodegradation of TCC has been shown to mitigate these effects in zebrafish by restoring microbial balance and reducing pathogen abundance [19]. In black-spotted frogs (*P. nigromaculatus*), TCC exposure perturbs the intestinal microbiota and triggers lipopolysaccharide (LPS) release, an endotoxin that enters the bloodstream and induces systemic toxicity via the gut-liver axis [20].

The black tiger shrimp *P. monodon* is a major aquaculture species in southern China with significant economic and ecological value [21]. The *P. monodon* is a shrimp species of the Decapoda, Penaeidae, and *Penaeus*. It is characterized by large size, omnivorous diet, delicious flesh, and rapid growth, making it one of the world’s top three farmed shrimp. The *P. monodon* exhibits a wide adaptability to salinity and can survive in diverse environments such as estuaries and marine habitats. Shrimp farming environments are susceptible to contamination from coastal water pollutants. TCC has been documented to exhibit significant toxicity toward various shrimp species. For instance, Fan et al. (2019) acutely exposed the oriental river prawn (*Macrobrachium nipponense*) (0.07 ± 0.02 g) to TCC at concentrations of 159.4, 223.1, 312.4, 437.3, and 612.2 μg/L for 96 h, and obtained a 96 h median lethal concentration (96 h LC_50_) of 261.6 μg/L [22]. For the mysid shrimp (*Mysidopsis bahia*), the 48 h and 96 h LC_50_ values were determined to be 15 and 100 μg/L, respectively [23]. TCC can disrupt the proper homeostatic regulation of gill physiology in *P. monodon* [10]. Currently, the toxicity of TCC has been investigated on fish and amphibians, but its effects on shrimp intestinal microbiota have not been investigated.

Conclude with a clear goal and hypothesis, we hypothesize that the environmental levels of TCC interfere with the diversity and functional stability of intestinal microbiota in *P. monodon* and cause possible dysbiosis. Therefore, this study utilized *P. monodon* as a toxicological model, exposing them to different concentrations of TCC for 14 days. High-throughput sequencing of the 16S rDNA gene was employed to analyze the perturbation of the intestinal microbiota’s structure and function. The findings are expected to offer a scientific evidence for ecological risk assessment of TCC and theoretical support for health management and pollution prevention strategies in shrimp aquaculture.

## 2. Materials and Methods

### 2.1. Chemicals

The TCC used in the present study was commercially sourced from Shanghai Yuanye Biotechnology Co., Ltd (Shanghai, China). with a purity of 98%. Before the exposure experiment, an appropriate amount of TCC was dissolved in a small volume of DMSO, and a stock solution of TCC at 100 mg/L was prepared and maintained in the dark at 4 °C. To eliminate the interference of cosolvents on the experimental results, the control received equivalent DMSO.

### 2.2. Shrimp and Rearing Conditions

Healthy *P. monodon* juveniles (6.5 ± 0.3 g) for this study were obtained from an industrialized indoor aquaculture facility in Shenzhen, China. All the shrimp were pathogen-free, exhibited vigorous activity, and showed no abnormalities in appearance or internal organs. Prior to the initiation of the stress experiment, the shrimp were held for a 7-day acclimation period in tanks containing 200 L of seawater, with 45 individuals per tank. The culture conditions during acclimatization included continuous aeration for 24 h, water temperature 29 ± 0.5 °C, pH 7.6–7.8, and salinity 30. The culture water was entirely replaced on a daily basis. Water quality parameters, including salinity, temperature, dissolved oxygen, pH, ammonia, and nitrite, were monitored throughout both the acclimation and exposure periods, and all were maintained within safe ranges for the shrimp. The shrimp were fed a commercial compound diet (Yuequn, Jieyang, China) at a daily ration of 5% of their body weight, with the amount adjusted based on consumption; residual feed and feces were promptly removed by siphoning thereafter.

### 2.3. TCC Exposure Experiment and Sampling

Following a one-week acclimation period, the shrimp were randomly allocated to three experimental groups: a control (CK) group, a 1 µg/L TCC (T1) group, and a 10 µg/L TCC (T10) group, with each group comprising three replicate tanks (45 shrimp per tank). Based on the previous studies, the environmentally relevant concentrations of TCC in aquatic systems range from 0.34 to 6.75 µg/L [4,5,6]; the 96 h LC_50_ of TCC for *M. nipponense* was reported as 261.6 µg/L [22]; and exposure to TCC at 0.1 and 6.3 µg/L for 96 h was shown to affect the survival, development, and physiological indicators of silver catfish (*R. quelen*) [7]. Therefore, we conducted a preliminary experiment and determined the TCC exposure concentrations for this study to be 1 and 10 µg/L. The CK group was cultured in normal TCC-free seawater. For the T1 group, the TCC concentration in the culture water was adjusted to 1 µg/L using the TCC stock solution. For the T10 group, the TCC concentration in the culture water was 10 µg/L, also regulated by the TCC stock solution. To maintain the nominal exposure concentrations, a complete water renewal was performed daily with the corresponding TCC dose replenished in each tank. The experimental groups differed only in their TCC exposure levels, all other culture conditions in each group were consistent with those during the acclimation period. Following the 14-day stress experiment, intestinal samples were obtained. To minimize individual variations among the shrimp, the intestines of 5 shrimp in each tank were randomly sampled and pooled together, then stored frozen at −80 °C for subsequent microbial community analysis. Intestinal microbiota samples from the three tanks in each group were individually subjected to high-throughput sequencing and bioinformatic analysis.

### 2.4. 16S rDNA High-Throughput Sequencing of Intestinal Microbiota

Total genomic DNA was extracted from intestinal microbiota samples using the FastDNA SPIN Kit for Feces (MP Biomedicals, Santa Ana, CA, USA) as per the manufacturer’s protocol, and its concentration and purity were subsequently determined by 1% agarose gel electrophoresis. The V4 region of the 16S rDNA gene was amplified with barcoded primers 515F (GTGCCAGCMGCCGCGG) and 806R (GGACTACHVGGGTWTCTAAT). The PCR amplifications were conducted in a final volume of 30 μL, with each reaction containing 10 ng of template DNA, 15 μL of Phusion^®^ High-Fidelity PCR Master Mix (New England Biolabs, Ipswich, MA, USA), and 0.2 μM of each forward and reverse primer. The amplification protocol consisted of 98 °C for 1 min; followed by 30 cycles of 98 °C for 10 s, 50 °C for 30 s, and 72 °C for 1 min; with a final step at 72 °C for 5 min. Successful amplification was confirmed through agarose gel electrophoresis analysis. The resulting PCR amplicons were combined in equimolar ratios and subsequently purified with the GeneJET Gel Extraction Kit (Thermo Scientific, Shanghai, China). The sequencing libraries were prepared using the NEB Next^®^ Ultra™ DNA Library Prep Kit for Illumina (New England Biolabs, Ipswich, MA, USA) in accordance with the manufacturer’s protocol. Library quality assessment was performed using a Qubit^®^ 2.0 Fluorometer (Thermo Scientific, Shanghai, China) alongside the Agilent Bioanalyzer 2100 system. Ultimately, paired-end sequencing (PE250) was conducted on the Illumina NovaSeq platform.

### 2.5. Bioinformatics Analysis

The raw reads obtained through sequencing were subjected to quality control, and the clean reads were obtained for subsequent analysis. Paired-end reads were assembled with FLASH and subsequently demultiplexed according to their unique barcodes. Chimeric sequences were detected and filtered out using the UCHIME algorithm [24]. Sequence analysis was performed with UPARSE v10 to cluster operational taxonomic units (OTUs) at a 97% similarity threshold [25]. Alpha diversity was assessed with Mothur v1.30.1 based on four indices: ACE, Chao1, Shannon, and Simpson, and the rarefaction curves were generated accordingly. A Venn diagram was created to visualize the number of unique and shared OTUs across different groups. Beta diversity was evaluated using principal coordinate analysis (PCoA). Bar plots illustrating microbial community composition at the phylum level were generated, and a heatmap focusing on the differential bacterial genera was produced using the heatmap function from the R version 3.4.4 pheatmap v1.0.12 software. Linear discriminant analysis effect size (LEfSe) was performed with the Python 2.7.18 LEfSe package v1.0 software to identify biomarkers quantifying differences among groups, and statistical significance was set at *p* < 0.05. A correlation network of intestinal bacteria was constructed at the phylum and genus levels using Cytoscape (http://www.cytoscape.org/, accessed on 16 September 2022); inter-species associations were determined by Spearman’s correlation and assessed for significance by Fisher’s test, with criteria set at |R| > 0.6 and *p* < 0.05. Changes in metabolic pathways were analyzed using the randomForest package v4.6-14 in R version 3.4.4 software according to the Kyoto Encyclopedia of Genes and Genomes (KEGG) database, and the functional prediction was performed using Python 3.8.19 PICRUSt2 v2.6.2 software, and statistical significance was *p* < 0.05.

### 2.6. Statistical Analysis

The data of α-diversity and bacterial relative abundance are presented as mean ± SE. Statistical significance was determined using one-way ANOVA and Kruskal–Wallis test with SPSS ver 21.0 software. The variance analysis of beta-diversity was performed using permutational multivariate analysis of variance (PERMANOVA). A *p*-value of less than 0.05 was defined as the threshold for statistical significance.

## 3. Results

### 3.1. Changes in Intestinal Microbial Diversity

**Figure 1:**
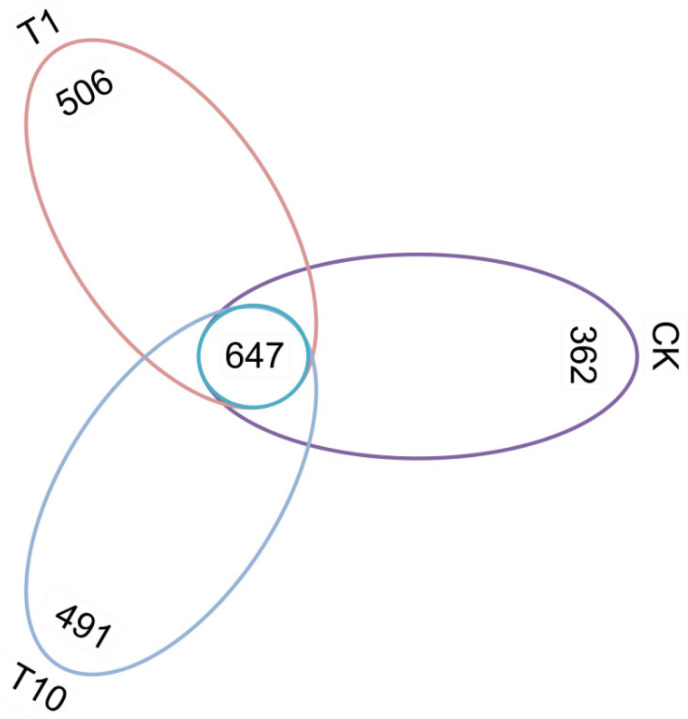
Venn analysis of the OTUs of the intestinal microbiota of *P. monodon* after TCC exposure. CK: Control group; T1: 1 μg/L TCC exposure group; T10: 10 μg/L TCC exposure group.

**Figure 2:**
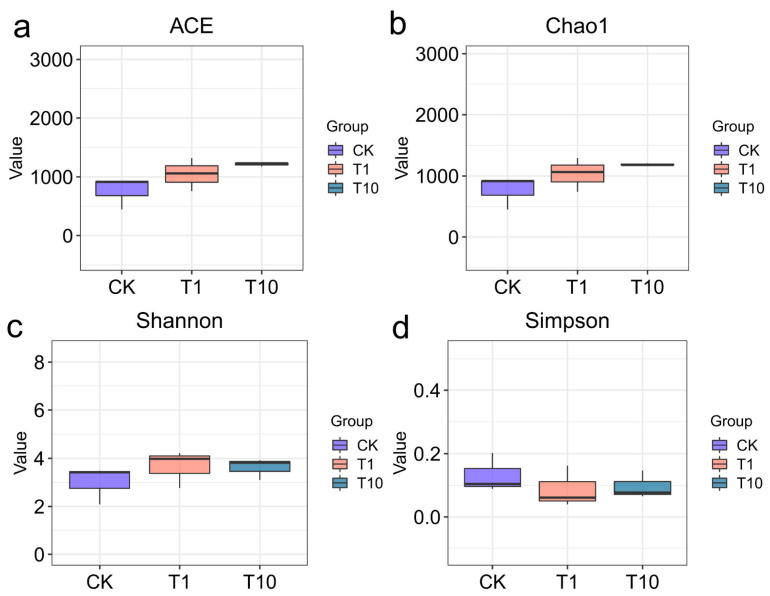
Changes in the α-diversity of the intestinal microbiota in *P. monodon* after TCC exposure. (**a**) ACE; (**b**) Chao1; (**c**) Shannon; (**d**) Simpson. CK: Control group; T1: 1 μg/L TCC exposure group; T10: 10 μg/L TCC exposure group.

**Figure 3:**
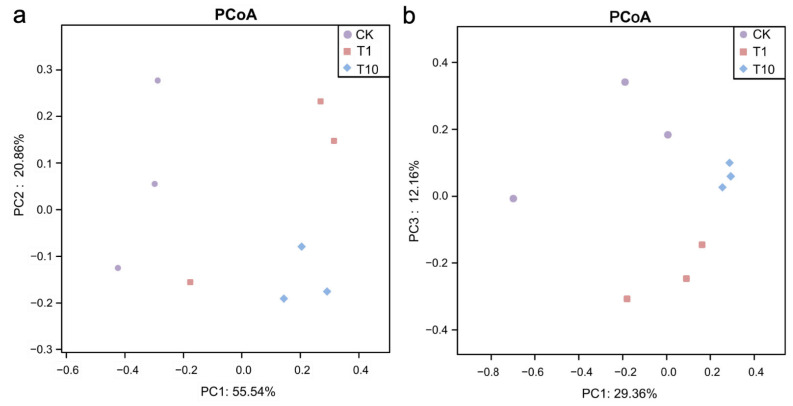
Changes in the β-diversity of the intestinal microbiota in *P. monodon* after TCC exposure based on the PCoA plot. (**a**) Weighted analysis of PCoA. (**b**) Unweighted analysis of PCoA. CK: Control group; T1: 1 μg/L TCC exposure group; T10: 10 μg/L TCC exposure group.

A core set of 647 OTUs was common to all three groups. Notably, both the T1 and T10 groups exhibited a greater number of unique OTUs compared to the CK group, with the T1 group displaying the highest count (Figure 1). Compared with the CK group, regarding intestinal microbial α-diversity, the ACE, Chao1, and Shannon indices were elevated in the T1 and T10 groups, whereas the Simpson index was reduced, but no statistically significant alterations were observed (*p* > 0.05) (Figure 2a–d, Table S1). The *β*-diversity of intestinal microbiota was analyzed based on PCoA, which showed that the microbial community patterns of the T1 and T10 groups were clearly separated from that of the CK group (Figure 3). The PERMANOVA further revealed a distinct separation in β-diversity among the groups, indicating significant differences (*p* < 0.05) in intestinal microbiota composition (Table S2).

### 3.2. Changes in the Composition of Intestinal Microbiota

**Figure 4:**
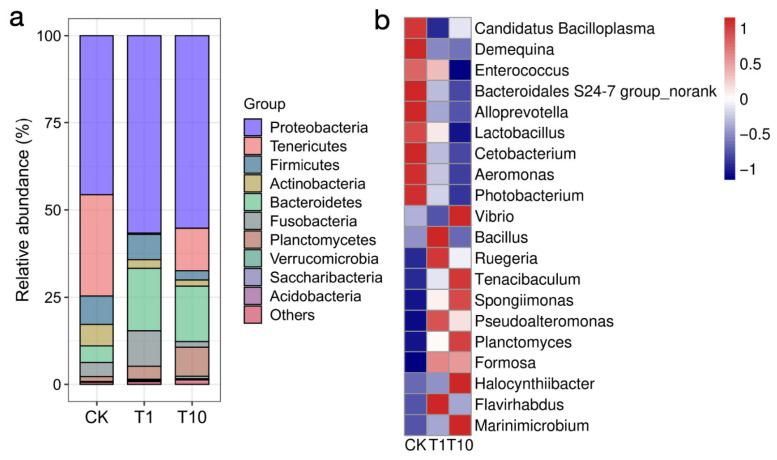
Alterations in the intestinal microbiota composition of *P. monodon* following TCC exposure. (**a**) Bacterial phyla. (**b**) Bacterial genera. The heatmap values represent scaled relative abundance, where positive and negative values correspond to an increase or decrease in abundance, respectively, and 0 indicates no change relative to the control group (CK). CK: Control group; T1: 1 μg/L TCC exposure group; T10: 10 μg/L TCC exposure group.

We characterized the structural changes in the intestinal microbial community by analyzing its composition at both the phylum and genus levels. At the phylum level (Figure 4a, Table S3), compared with the CK group, the relative abundances of Bacteroidetes was significantly increased in the T1 and T10 groups (*p* < 0.05); the abundance of Planctomycetes was increased in both groups, but only significant in the T10 group (*p* < 0.05); the abundance of Proteobacteria was increased in both groups without statistical significance (*p* > 0.05). However, the abundance of Tenericutes was significantly decreased in the T1 and T10 groups (*p* < 0.05); the abundance of Actinobacteria was decreased in both groups, but only significant in the T10 group (*p* < 0.05); the abundance of Firmicutes was decreased in both groups without statistical significance (*p* > 0.05).

At the genus level (Figure 4b, Table S4), the relative abundances of *Formosa* and *Marinimicrobium* were significantly increased in the T1 and T10 groups relative to the CK group, but *Candidatus Bacilloplasma* was significantly decreased (*p* < 0.05); the abundances of *Flavirhabdus*, *Planctomyces*, and *Halocynthiibacter* were increased in the T1 and T10 groups, but *Flavirhabdus* only significant in the T1 group, *Planctomyces* and *Halocynthiibacter* only significant in the T10 group (*p* < 0.05). Additionally, the relative abundances of *Ruegeria*, *Tenacibaculum*, *Spongiimonas*, and *Pseudoalteromonas* were increased in the T1 and T10 groups, while *Demequina*, *Enterococcus*, *Cetobacterium*, *Photobacterium*, *Aeromonas*, *Bacteroidales S24-7 group_norank* (“norank” refers to bacteria that have not been fully classified), *Alloprevotella*, and *Lactobacillus* were decreased, but none of them were statistically significant. The relative abundance of *Bacillus* was elevated in the T1 group, while a decrease was noted in the T10 group; in contrast, *Vibrio* was decreased in the T1 group but increased in the T10 group; but none of them were statistically significant. Furthermore, the changes in the top 10 bacterial genera were also analyzed, and the results indicated that only *Candidatus Bacilloplasma* showed significant alterations in the two exposure groups (Table S5).

### 3.3. Identification of Key Biomarkers of Intestinal Microbiota

**Figure 5:**
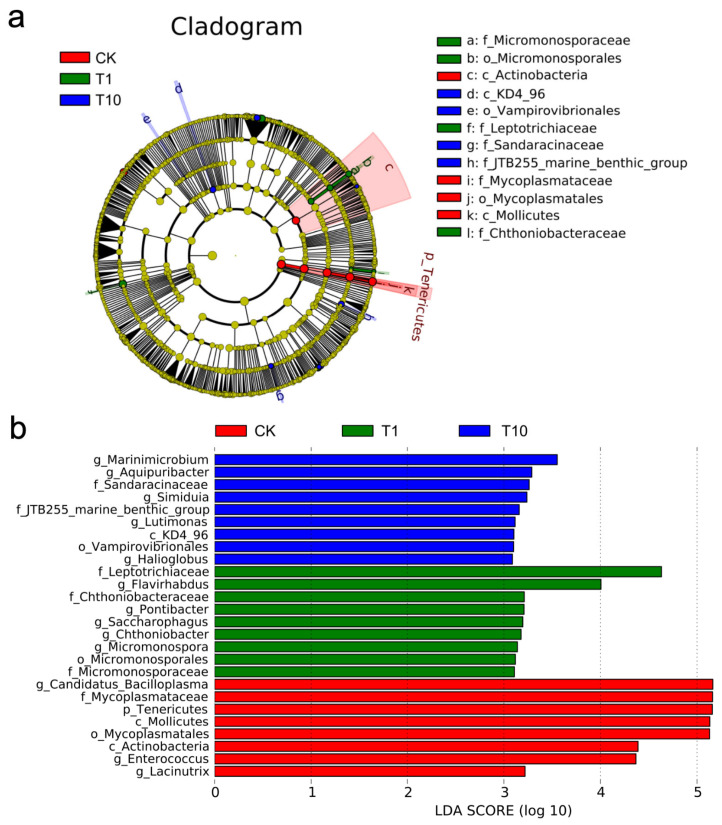
Intergroup differences in the intestinal microbiota in *P. monodon* after TCC exposure. (**a**) LEfSe cladogram. (**b**) LDA score. CK: Control group; T1: 1 μg/L TCC exposure group; T10: 10 μg/L TCC exposure group.

The differential bacterial taxa in the intestinal microbiota associated with exposure response were identified via LEfSe analysis. On the basis of the cladogram, the family Mycoplasmataceae was identified as the dominant taxon in the CK group (*p* < 0.05); the families Micromonosporaceae, Leptotrichiaceae, and Chthoniobacteraceae were dominant in the T1 group (*p* < 0.05); the families Sandaracinaceae and JTB255-marine-benthic-group were dominant in the T10 group (*p* < 0.05) (Figure 5a). Differential bacterial across groups begin to emerge when the LDA score exceeds 3.0, so this threshold was chosen to define significant biomarkers. Notably, the genera *Lacinutrix*, *Enterococcus*, and *Candidatus_Bacilloplasma* were dominant in the CK group (*p* < 0.05); the genera *Micromonospora*, *Chthoniobacter*, *Saccharophagus*, *Pontibacter*, and *Flavirhabdus* were dominant in the T1 group (*p* < 0.05); the genera *Halioglobus*, *Lutimonas*, *Simiduia*, *Aquipuribacter*, and *Marinimicrobium* were dominant in the T10 group (*p* < 0.05) (Figure 5b).

### 3.4. Network Relationships Among Intestinal Bacteria

**Figure 6:**
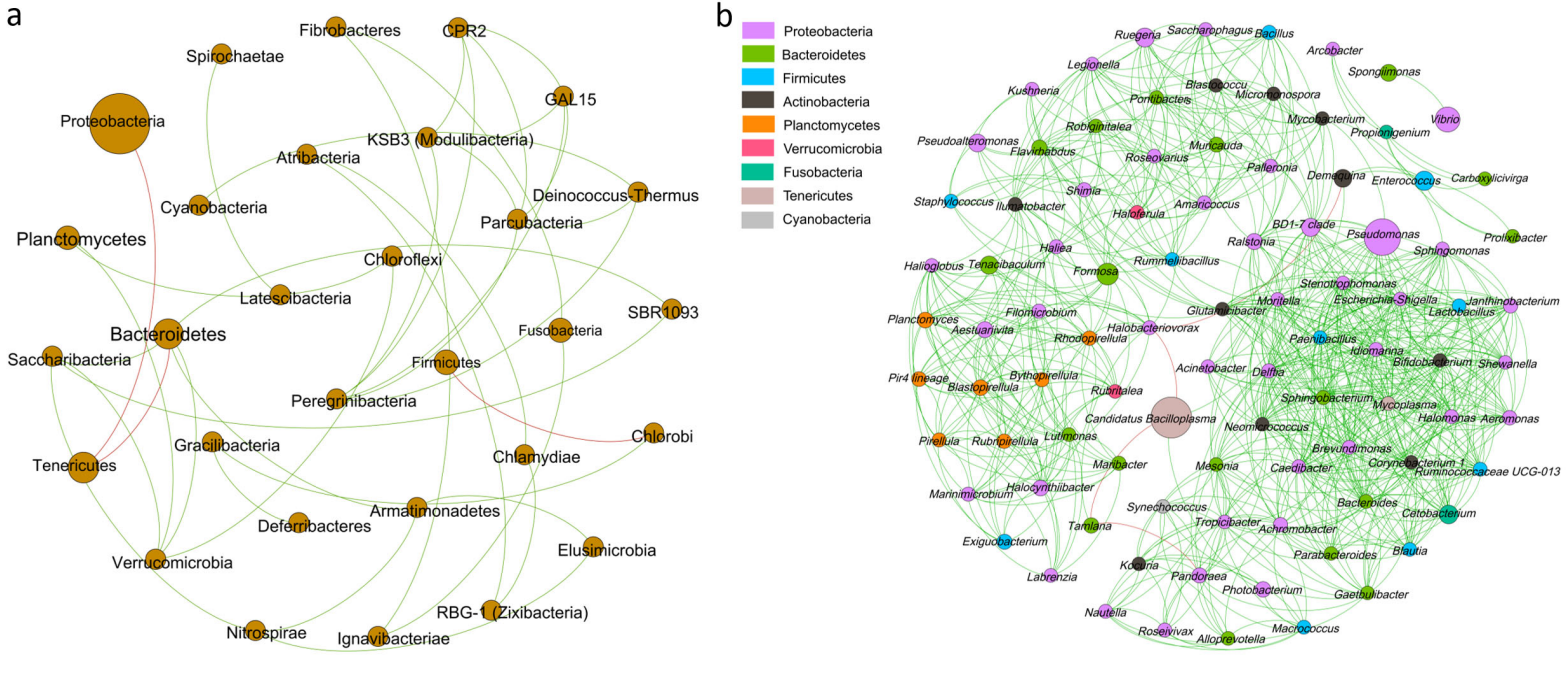
The correlation network of the intestinal microbiota in *P. monodon* after TCC exposure. (**a**) Phylum-level correlation network. (**b**) Genus-level correlation network. Circular nodes represent bacterial phyla or genera. The size of each node is proportional to its relative abundance. The lines connecting the nodes represent the presence of correlations between them, green and red lines corresponding to positive and negative correlations, respectively. CK: Control group; T1: 1 μg/L TCC exposure group; T10: 10 μg/L TCC exposure group.

The relationships among intestinal microbiota were explored at the phylum and genus levels, respectively. At the phylum level, both Proteobacteria and Bacteroidetes showed a significantly negative correlation with Tenericutes (*p* < 0.05); Planctomycetes exhibited a significantly positive correlation with Chloroflexi and Verrucomicrobia, respectively (*p* < 0.05) (Figure 6a). Furthermore, the genus *Vibrio* showed a positive correlation with *Spongiimonas*; *Photobacterium* showed positive correlations with *Candidatus Bacilloplasma*, *Macrococcus*, and *Kocuria*, respectively (*p* < 0.05); *Aeromonas* showed positive correlations with *Pseudomonas*, *Lactobacillus*, *Cetobacterium*, *Sphingomonas*, and *Bifidobacterium*, respectively (*p* < 0.05) (Figure 6b).

### 3.5. Alterations in the Intestinal Microbiota Metabolic Function

**Figure 7:**
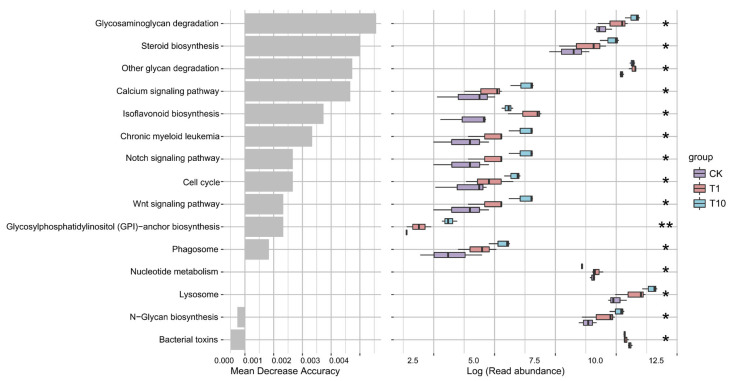
The top 20 metabolic functions with significant changes in the intestinal microbiota in *P. monodon* after TCC exposure. Statistically significant differences are indicated by asterisks (* *p* < 0.05, ** *p* < 0.01). CK: Control group; T1: 1 μg/L TCC exposure group; T10: 10 μg/L TCC exposure group.

Based on predictive analysis, the metabolic potential of the intestinal microbiota was assessed. Based on functional prediction, several metabolic pathways were significantly altered in the T1 and T10 groups compared to the CK group. Specifically, the “glycosaminoglycan degradation”, “other glycan degradation”, “N-glycan biosynthesis”, “steroid biosynthesis”, “isoflavonoid biosynthesis”, and “nucleotide metabolism” pathways were markedly enhanced. In contrast, the “bacterial toxins” pathway was significantly reduced (Figure 7).

## 4. Discussion

TCC as a widely used antimicrobial agent, poses a non-negligible threat due to its persistent residues and bioaccumulation in aquatic environments. In this study, following a 14-day exposure to 1 and 10 μg/L of TCC, the cumulative survival rates of *P. monodon* were 85.56% and 75.56%, respectively, both significantly lower than the control, demonstrating TCC’s toxicity to this shrimp species (Figure S1). Notably, the intestinal microbiota of aquatic animals acts as a critical biological barrier, playing a vital role not only in host health but also in the organism’s response to environmental stressors. Therefore, the stability of the intestinal microecology under pollutant stress is directly linked to the host’s health status and environmental adaptability. Base on this background, investigating how TCC exposure affects the intestinal microbiota of the tiger shrimp *P. monodon*, an economically important species, is essential for a deeper understanding of TCC’s toxic mechanisms and for assessing its ecological risks.

Intestinal microbial diversity is crucial for maintaining host health, ecosystem stability, and coping with environmental stress [11,26]. In this study, the increases in the ACE, Chao1, and Shannon indices, along with the decrease in the Simpson index, indicated that TCC exposure altered the diversity of the intestinal microbiota in the shrimp. It likely inhibited the original dominant bacterial populations or created ecological niches for some rare or tolerant species, thereby reducing the dominance of a few species and making the community structure more homogeneous and diverse. This suggested that TCC exposure drove the microbial community toward a direction with greater species richness and more balanced distribution of individuals, which could further affect the stability of the community’s ecological functions. However, previous studies have shown that the zebrafish exposed to 100 and 1000 μg/L TCC for 21 days exhibited a decrease in intestinal microbial diversity [17], which differed from our findings. Zebrafish are vertebrates and were reared in a relatively simple environment; in contrast, shrimp are invertebrates and economically important aquaculture species, and their rearing environment was relatively complex. Additionally, in this study, the shrimp were exposed to environmentally relevant concentrations of TCC (1 and 10 μg/L) for 14 d, resulting in a lower stress level compared to that of the zebrafish in the previous studies. It can be concluded that the differences in the responses of different species to pollutants were associated with variations in their intestinal physiological structure, basic microbiota composition, as well as the exposure concentration and duration.

TCC exposure led to a disturbance in the compositional homeostasis of the intestinal microbiota. Proteobacteria include a variety of Gram-negative bacteria, some of which are opportunistic pathogens and usually associated with dysbiosis and intestinal inflammation [27]. In this study, the increased of Proteobacteria indicated that TCC exposure might promote the proliferation of potential pathogens, thereby increasing the risk of intestinal infection and inflammation in shrimp. Bacteroidetes mainly decompose indigestible polysaccharides and yield short-chain fatty acids (SCFAs), which are implicated in the regulation of immunity and intestinal health [28]. Planctomycetes are known as an important source of bioactive molecules [29]. In this study, TCC exposure induced an increase in Bacteroidetes and Planctomycetes in the shrimp intestine, which likely represented an adaptive response of the intestinal microbiota to environmental stress. The rise in Bacteroidetes suggested a potential activation of metabolic pathways involved in complex polysaccharide degradation and SCFAs production, which might have enhanced the host’s nutrient acquisition capacity and influenced the intestinal immune environment. Concurrently, the increase in Planctomycetes indicated a potential elevation in the synthesis potential of bioactive substances, thereby indirectly supporting the maintenance of intestinal health and physiological homeostasis in shrimp. Firmicutes are involved in SCFAs production and energy absorption [30]. Actinobacteria can produce substances with immunomodulatory and antibacterial activities [31]. In this study, the decrease in Firmicutes and Actinobacteria indicated that TCC exposure disturbed the homeostasis of functional bacteria in the shrimp intestine, disrupted the intestinal microecology, which was not conducive to the maintenance of normal intestinal physiological functions in shrimp. Tenericutes are major component of the intestinal microbiota of shrimp [32]. Tenericutes are major component of the intestinal microbiota of shrimp [32]. In this study, the observed decline of Tenericutes in the shrimp intestines demonstrated that TCC exposure specifically suppressed core symbiotic bacterial communities. As a distinctive bacterial phylum characterized by the absence of cell walls, Tenericutes typically established close symbiotic or parasitic relationships with the host. The reduction in their abundance not only potentially compromised the metabolic network of the intestinal microbiota, but more critically, likely disrupted the homeostatic balance maintained with the host’s immune system, thereby diminishing the intestine’s physiological resilience to environmental stressors. After 21 days of exposure to 100 and 1000 μg/L TCC, the relative abundance of Fusobacteriota was increased in zebrafish, while that of Proteobacteria and Actinobacteria were decreased [17]. This finding is not entirely consistent with the results of the present study, reaffirming that the impact of TCC on the intestinal microbiota of aquatic animals is closely associated with species, pollutant concentration, and exposure duration.

The homeostasis of some potential bacteria closely associated with host health in the shrimp intestine was also affected by TCC exposure. *Candidatus Bacilloplasma*, a predominant bacterium in the intestines of shrimp, often undergoes dynamic shifts during disease outbreaks, which may consequently impact the host’s health [33]. In this study, the decreased abundance of *Candidatus Bacilloplasma* suggests that TCC exposure may alter the intestinal microecology of shrimp, thereby inhibiting the proliferation of this bacterium. As functionally active substance-producing bacteria, *Alloprevotella*, *Bacteroidales S24-7 group* and *Cetobacterium* can metabolize carbohydrates to produce SCFAs [34,35,36]; *Lactobacillus* and *Enterococcus* are commonly used probiotics in aquaculture [37,38]; *Demequina* can produce α-amylase to decompose starch [39]. In this study, TCC exposure caused a decrease in the above-mentioned potential beneficial bacteria, which indicated that the core beneficial functional microbiota responsible for SCFAs production, starch decomposition, and probiotic effects in the intestine was generally weakened. This collective functional impairment consequently compromised the microbial community’s ability to maintain the nutritional metabolism and health homeostasis the host. *Pseudoalteromonas* as a beneficial bacterium can enhance the shrimp’s resistance to *Vibrio* infection [40]. *Formosa* are capable of degrading algal polysaccharides [41]. In this study, the decrease in *Pseudoalteromonas* and *Formosa* might be a positive response to TCC exposure, aiming to alleviate the stress induced by TCC in the shrimp intestine. *Vibrio*, *Photobacterium*, *Aeromonas* and *Tenacibaculum* are several common pathogenic bacteria in shrimp aquaculture [42,43,44,45]. In this study, following TCC exposure, the abundance of *Photobacterium* and *Aeromonas* were decreased but *Tenacibaculum* was increased in the shrimp intestine, while that of *Vibrio* was decreased under low-dose exposure but increased at high doses, indicating that TCC exposure also disrupted the homeostasis of harmful bacteria. Based on these findings, TCC exposure not only disturbed the abundance of detrimental bacterial populations in the shrimp intestine but also affected the abundance of beneficial bacteria. This might have been a general form of intestinal dysbiosis or disruption, which altered the microenvironment within the intestine, thereby breaking the original balance among the intestinal microbiota and driving the remodeling of the microbial community structure. It is noteworthy that in our previous study, TCC exposure was found to induce oxidative stress, reduce antioxidant capacity, and alter the transcription of genes involved in stress response, apoptosis, detoxification, and osmoregulation in *P. monodon* [10]. Therefore, in the present study, the intestinal microbial shifts observed in shrimp under TCC exposure might represent a secondary response to systemic stress in the host. These systemic physiological stress responses likely indirectly affected the microenvironment of the intestine, thereby in turn results in the structural reshaping of the microbial community.

Based on our findings, TCC exposure markedly influenced the metabolic potential of the intestinal microbiota of the shrimp. For instance, after TCC exposure, the functions such as “glycosaminoglycan degradation”, “other glycan degradation”, “N-glycan biosynthesis”, “steroid biosynthesis”, “isoflavonoid biosynthesis”, and “nucleotide metabolism” were all enhanced. These changes suggested that TCC exposure might affect the microbial activity related to carbon source utilization, energy metabolism, and the synthesis of secondary metabolites. The degradation of complex carbohydrates such as glycosaminoglycans by intestinal microbiota can produce immunomodulatory oligosaccharides and short-chain fatty acids, which might enhance nutrient absorption efficiency and activate the shrimp’s innate immune response. Meanwhile, microbial-derived steroids might provide essential nutritional supplementation for shrimp, particularly under stressful conditions, helping to maintain cell membrane structure and normal physiological functions. On the other hand, the decrease in “bacterial toxins”-related functions indicated that TCC exposure might reduce the toxin-producing ability of some potential pathogenic bacteria. This reduction could alleviate direct toxic damage to the shrimp intestine, indicating that TCC exposure might modulate microbial pathogenicity to some extent. In general, these changes in metabolic functions indicated a stress response or microbial imbalance of the intestinal microbiota under TCC exposure. However, it is important to note that these inferred changes in microbial function rely solely on KEGG predictions and lack support from quantitative or experimental assays. Therefore, future studies integrating techniques such as metagenomics and metabolomics are required to validate these findings.

It is important to note a key limitation of our study design. For example, the assessment of the intestinal microbiota at only a single terminal time point. While this provides a snapshot of the microbial community after 14 days of exposure, it does not allow us to capture the temporal dynamics, such as the progression of dysbiosis or the potential for early recovery. Therefore, our conclusions regarding the effects of TCC are specific to this exposure duration. Future studies incorporating multiple time points, including a pre-exposure baseline, would be invaluable for elucidating the complete trajectory and resilience of the microbial communities in response to TCC. In addition, we pooled intestines from multiple shrimp for sequencing. While this method is acceptable, it may mask individual variability in the microbial community. Future studies could consider analyzing intestinal microbiota from individual specimens with increased sample size to better characterize these changes. Another limitation is the lack of data on survival and growth performance of the shrimp during the 14-day exposure period. These metrics would be very relevant to understanding the potential biological impact of TCC exposure and should be addressed in future studies.

## 5. Conclusions

This study provided the first evidence of the toxic effects of an antimicrobial contaminant TCC exposure on the intestinal microbiota homeostasis of *P. monodon*. Our findings demonstrated that TCC exposure reshaped the intestinal microbial ecosystem, which was characterized by an increase in diversity and distinct compositional shifts. Notably, these alterations were not merely structural but extended to functional changes, as significant enrichment was observed in key microbial metabolic pathways, including those related to glycan biosynthesis and degradation, steroid and isoflavone biosynthesis, and nucleotide metabolism. The observed decline in potential pathogens (*Photobacterium* and *Aeromonas*) alongside beneficial genera (*Alloprevotella*, *Bacteroidales S24-7 group_norank*, *Cetobacterium*, *Enterococcus*, and *Lactobacillus*) highlighted the complex ecological impact of TCC. Collectively, the findings of this study highlight the importance of monitoring TCC contamination and improving water quality in shrimp aquaculture. Additionally, TCC-sensitive intestinal bacterial biomarkers can be identified for environmental monitoring and assessment, while intestinal microbiota characteristics could potentially be utilized to enhance shrimp defense capabilities against TCC exposure.

## Data Availability

Data will be made available upon request.

## Supplementary Materials

The following supporting information can be downloaded at: https://www.mdpi.com/article/10.3390/biology14111542/s1. Table S1: Changes in the α-diversity of the intestinal microbiota of *P. monodon* after TCC exposure; Table S2: PERMANOVA of the intestinal microbiota β-diversity based on PCoA; Table S3: Changes in the relative abundances of the intestinal bacterial phyla of *P. monodon* after TCC exposure; Table S4: Changes in the relative abundances of the intestinal bacterial genera of *P. monodon* after TCC exposure; Table S5: Changes in the relative abundances of the top 10 of the intestinal bacterial genera of *P. monodon* after TCC exposure; Figure S1: Cumulative survival rate of *P. monodon* after 14 days of TCC exposure.
