# Supplementary material for: Changes in Intestinal Microbial Community of the Black Tiger Shrimp Penaeus monodon in Response to Triclocarban Exposure
Source: Biology (Basel). 2025 Nov 3;14(11):1542. doi: 10.3390/biology14111542 (PMC12650320; doi:10.3390/biology14111542)
Supplement: Supplementary file 1 [file biology-14-01542-s001.zip › biology-3921100-supplementary.pdf]

Supplementary Materials

Table S1. Changes in the α-diversity of the intestinal microbiota of *P. monodon* after TCC exposure. Different letters indicate significant differences in the same indicator between groups ($P < 0.05$), while the same letters show no significant differences ($P > 0.05$).

Group	CK	T1	T10
ACE	762.67 ± 157.35[a]	1045.67 ± 162.11[a]	1214.00 ± 19.04[a]
Chao1	764.67 ± 154.84[a]	1033.00 ± 159.24[a]	1189.33 ± 8.84[a]
Shannon	3.00 ± 0.45[a]	3.66 ± 0.45[a]	3.61 ± 0.26[a]
Simpson	0.13 ± 0.04[a]	0.09 ± 0.04[a]	0.10 ± 0.03[a]

Table S2. PERMANOVA of the intestinal microbiota β-diversity based on PCoA.

Method name	Weighted.permanova	Unweighted.permanova
Test statistic name	pseudo-F	pseudo-F
Sample size	9	9
Number of groups	3	3
Test statistic	3.03	1.64
P-value	0.023	0.008
Number of permutations	999	999

Table S3. Changes in the relative abundances of the intestinal bacterial phyla of *P. monodon* after TCC exposure. Different letters indicate significant differences in the same indicator between groups ($P < 0.05$), while the same letters show no significant differences ($P > 0.05$).

Group	CK	T1	T10
Proteobacteria	45.66 ± 7.32[a]	56.64 ± 5.36[a]	55.26 ± 7.45[a]
Tenericutes	28.97 ± 6.68[b]	0.36 ± 0.09[a]	12.14 ± 3.05[a]
Firmicutes	8.11 ± 2.71[a]	7.24 ± 2.35[a]	2.64 ± 0.96[a]
Actinobacteria	6.27 ± 1.87[b]	2.46 ± 0.48[ab]	1.75 ± 0.39[a]
Bacteroidetes	4.74 ± 0.64[a]	17.99 ± 4.33[b]	15.98 ± 0.91[b]
Fusobacteria	4.06 ± 2.20[a]	10.12 ± 7.29[a]	1.60 ± 0.93[a]
Planctomycetes	1.39 ± 0.67[b]	3.72 ± 1.45[ab]	8.30 ± 2.39[a]
Verrucomicrobia	0.07 ± 0.04[a]	0.32 ± 0.14[ab]	0.70 ± 0.22[b]
Saccharibacteria	0.06 ± 0.03[a]	0.25 ± 0.11[a]	0.20 ± 0.03[a]
Acidobacteria	0.08 ± 0.05[a]	0.11 ± 0.04[a]	0.12 ± 0.04[a]
Others	0.60 ± 0.25[a]	0.79 ± 0.16[a]	1.26 ± 0.20[a]

Table S4. Changes in the relative abundances of the intestinal bacterial genera of *P. monodon* after TCC exposure. Different letters indicate significant differences in the same indicator between groups ($P < 0.05$), while the same letters show no significant differences ($P > 0.05$).

Group	CK	T1	T10
Candidatus Bacilloplasma	28.77 ± 6.72[b]	0.13 ± 0.06[a]	11.99 ± 3.03[a]
Demequina	5.19 ± 2.34[a]	0.75 ± 0.27[a]	0.48 ± 0.14[a]
Enterococcus	4.86 ± 4.25[a]	3.66 ± 3.64[a]	0.00 ± 0.00[a]
Vibrio	4.69 ± 1.77[a]	3.55 ± 0.60[a]	8.67 ± 3.87[a]
Cetobacterium	3.77 ± 2.35[a]	2.15 ± 1.05[a]	1.60 ± 0.93[a]
Photobacterium	2.00 ± 0.98[a]	0.78 ± 0.40[a]	0.11 ± 0.04[a]
Ruegeria	1.30 ± 0.55[a]	4.17 ± 2.25[a]	2.61 ± 0.19[a]
Tenacibaculum	1.06 ± 0.87[a]	2.07 ± 1.06[a]	3.57 ± 0.66[a]
Spongiimonas	1.01 ± 0.69[a]	1.66 ± 0.46[a]	2.18 ± 1.09[a]
Pseudoalteromonas	0.89 ± 0.45[a]	3.11 ± 1.38[a]	2.24 ± 0.11[a]
Aeromonas	0.79 ± 0.62[a]	0.38 ± 0.17[a]	0.20 ± 0.15[a]
Bacteroidales S24-7 group_norank	0.36 ± 0.17[a]	0.21 ± 0.06[a]	0.15 ± 0.09[a]
Alloprevotella	0.26 ± 0.18[a]	0.09 ± 0.02[a]	0.05 ± 0.02[a]
Planctomyces	0.23 ± 0.12[a]	1.10 ± 0.42[ab]	1.86 ± 0.57[b]
Bacillus	0.17 ± 0.08[a]	0.94 ± 0.81[a]	0.08 ± 0.01[a]
Lactobacillus	0.15 ± 0.14[a]	0.11 ± 0.03[a]	0.06 ± 0.02[a]
Formosa	0.04 ± 0.02[a]	6.14 ± 2.22[b]	5.87 ± 0.99[b]
Halocynthiibacter	0.03 ± 0.01[a]	0.32 ± 0.14[a]	2.84 ± 0.77[b]
Flavirhabdus	0.02 ± 0.01[a]	2.06 ± 0.88[b]	0.43 ± 0.11[ab]
Marinimicrobium	0.00 ± 0.00[a]	0.14 ± 0.08[b]	0.73 ± 0.23[b]

Table S5. Changes in the relative abundances of the top 10 of the intestinal bacterial genera of *P. monodon* after TCC exposure. Different letters indicate significant differences in the same indicator between groups ($P < 0.05$), while the same letters show no significant differences ($P > 0.05$).

Group	CK	T1	T10
Candidatus Bacilloplasma	28.77 ± 6.72[b]	0.13 ± 0.06[a]	11.99 ± 3.03[a]
Pseudomonas	13.89 ± 10.08[a]	16.13 ± 6.75[a]	4.30 ± 1.55[a]
Alteromonadales_norank	11.19 ± 7.58[a]	13.14 ± 9.16[a]	22.97 ± 5.76[a]
Demequina	5.19 ± 2.34[a]	0.75 ± 0.27[a]	0.48 ± 0.14[a]
Enterococcus	4.86 ± 4.25[a]	3.66 ± 3.64[a]	0.00 ± 0.00[a]
Vibrio	4.69 ± 1.77[a]	3.55 ± 0.60[a]	8.67 ± 3.87[a]
Cetobacterium	3.77 ± 2.35[a]	2.15 ± 1.05[a]	1.60 ± 0.93[a]
BD1-7 clade	2.35 ± 1.33[a]	3.21 ± 1.35[a]	1.22 ± 0.56[a]
Photobacterium	2.00 ± 0.98[a]	0.78 ± 0.40[a]	0.11 ± 0.04[a]
Pandoraea	1.61 ± 0.83[a]	0.53 ± 0.16[a]	0.14 ± 0.04[a]

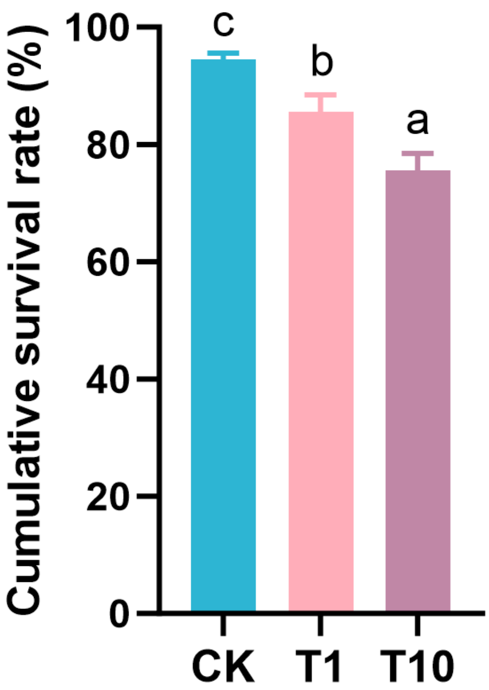

Figure S1. Cumulative survival rate of *P. monodon* after 14 days of TCC exposure.
